# Labor Augmentation with Oxytocin Decreases Glutathione Level

**DOI:** 10.1155/2009/807659

**Published:** 2009-04-16

**Authors:** Naomi Schneid-Kofman, Tali Silberstein, Oshra Saphier, Iris Shai, Dorith Tavor, Ariela Burg

**Affiliations:** ^1^Department of Obstetrics and Gynecology, Faculty of Health Sciences, Soroka University Medical Center, Ben-Gurion University of the Negev, Beer-sheva, Israel; ^2^Sansana 507, D.N. Negev3, 85334, Israel; ^3^Department of Chemical Engineering, Sami Shamoon College of Engineering, Ashdod, Israel; ^4^Department of Epidemiology, The S. Daniel Abraham International Center for Health and Nutrition, Ben-Gurion University, Beer-Sheva, Israel

## Abstract

*Objective*. To compare oxidative stress following spontaneous vaginal delivery with that induced by Oxytocin augmented delivery. *Methods*. 98 women recruited prior to labor. 57 delivered spontaneously, while 41 received Oxytocin for augmentation of labor. Complicated deliveries and high-risk pregnancies were excluded. Informed consent was documented. Arterial cord blood gases, levels of Hematocrit, Hemoglobin, and Bilirubin were studied. Glutathione (GSH) concentration was measured by a spectroscopic method. Plasma and red blood cell (RBC) levels of Malondialdehyde indicated lipid peroxidation. RBC uptake of phenol red denoted cell penetrability. SPSS data analysis was used. *Results*. Cord blood GSH was significantly lower in the Oxytocin group (2.3 ± 0.55 mM versus 2.55 ± 0.55 mM, *P* = .01). No differences were found in plasma or RBC levels of MDA or in uptake of Phenol red between the groups. *Conclusion*. Lower GSH levels following Oxytocin augmentation indicate an oxidative stress, though selected measures of oxidative stress demonstrate no cell damage.

## 1. Introduction

Labor is a state of stress, though the oxidative burden upon the fetus is of controversy in literature [1, 2]. Neonatal adverse outcome may result from oxidative stress, thus cord blood pH is currently the most used method for assessing fetal oxidative metabolic stress with some correlation to neonatal outcome [3, 4].

Reactive oxygen free radicals are produced by aerobic cell metabolic activity. The accumulation of these radicals can produce toxic changes within the cells by an uncontrolled self-enhancing process of lipid peroxidation of membranes and inner cell components resulting in a disruption of membrane lipids and other cell components. The cell defensive system consists of antioxidative free-radical scavenging molecules such as glutathione (GSH—a tripeptide consisting of glutamic acid-cysteine-glycine). GSH acts as the substrate for the enzyme glutathione peroxidase. As such it is an important component of intracellular antioxidant defense, protecting cytosolic organelles, in particular, from the damaging effects of hydroperoxides. In addition, GSH also acts synergistically with ascorbic acid and alphatocopherol to recycle these nutrient antioxidant vitamins to their reduced state after their interaction with reducing chemical species inside the cell [5].

Red blood cells (RBCs) are prone to lipid peroxidation by virtue of their function as oxygen carriers and their lipid composition. Measurement of Malondialdehyde (MDA) content using thiobarbituric reagent is widely used to quantify lipid peroxidation and is indicative of the amount of oxygen radicals in the RBC environment. An alternative method of evaluating lipid peroxidation in RBC is by measuring uptake of phenol red [6].

Initiation and progress of labor are generated by endogenous Oxytocin hormone levels. Low contraction frequency and protracted labor are treated with administration of exogenous Oxytocin for augmenting labor [7].

Uterine blood flow is reduced during contractions and further reduced during intensive uterine activity, resulting in compromised placental blood flow. Therefore, administration of Oxytocin during labor may induce fetal oxidative stress. Previous studies have found no adverse effect of Oxytocin treatment on pH levels [8], and Oxytocin augmentation did not increase perinatal risk [9]. Measuring the level of antioxidation enzymes in cord blood following Oxytocin treatment provides useful information regarding the extent of fetal oxidative stress and the information regarding the safe use of this treatment in labor.

In this study, we compared the oxidative stress induced by normal vaginal delivery with that following Oxytocin augmented delivery.

## 2. Materials and Methods

Prior to delivery, 98 women were recruited from labor and delivery department in Soroka medical center during November 2006 and February 2007. Of these, 57 delivered uncomplicated spontaneous vaginal deliveries; 41 received Oxytocin treatment (Oxytocin Injection, BP 10 Units/ampule, produced by Rotexmedica GmbH, Germany) for augmentation of labor resulting in vaginal delivery. Four women of the control group and one of the Oxytocin group were excluded during data analysis, all for incomprehensive lab analysis (hemolytic sample, coagulated sample). The protocol of treatment in our institution for Oxytocin administration was followed. Oxytocin was diluted at 5 mIU in 500 mL NaCl 0.9%, administered at an initial rate of 2 mIU/min and increased by 2 mIU every 20–40 minutes, until effective regular contractions were achieved. The maximum dose administered was 16 mIU/min. Dose and duration of therapy were documented. The final appropriate dose administered was determined by observed frequency of resulting uterine contractions and by progress of labor.

Excluded from the study were multifetal deliveries, surgical or mechanical deliveries, Postdate deliveries (42 completed weeks of pregnancy), or suspected intrauterine growth restricted fetuses.

Data were collected by personal interviews, validated through medical records. We collected data regarding demographics, past medical and obstetric history, the indication for augmentation of labor, duration of labor (first and second stages), neonatal data, and use of epidural anesthesia.

Blood samples were drawn from the umbilical cord artery immediately after fetal delivery, before delivery of the placenta and stored at 4°C, up to 8 hours. Whole blood samples were analyzed within five minutes of collection by a blood gas analyzer for pH, carbon dioxide (pCO_2_), oxygen (pO_2_), oxygen saturation, and base excess (BE). Levels of Hematocrit, Hemoglobin, and Bilirubin were analyzed within an hour of delivery. Measurements of oxidative stress indicators were concluded within eight hours. Glutathione concentration was determined by a spectroscopic method, measuring the production of the yellow anion produced by redox reaction between sulphahydryl (–SH) groups and the reagent 5,5′-Dithiobis-2-nitrobenzoic acid (DTNB) [10]. Plasma and red blood cell (RBC) levels of Malondialdehyde (MDA) were measured for lipid peroxidation; RBC uptake of phenol red was measured for cell penetrability [6]. Measurements of penetrability are displayed as arbitrary units of absorption.

Statistical analysis was performed with an SPSS software package (SPSS, Chicago, IL). Statistical significance was determined using the *χ* ^2^ test, the Fisher exact test for differences between qualitative variables, and the *t*-test for differences between continuous variables. Odds ratios (OR) and their 95% confidence intervals (CI) were calculated. Pearson correlation coefficient was used to calculate the correlation of GSH on pO_2_. Multivariate analysis was preformed. *P* < .05 was considered statistically significant. This study is preliminary in the field of Oxytocin in vaginal delivery and oxidative stress. Due to lack of previous data, power analysis was not done.

The study was approved by the Institutional Review Board; informed consent was documented.

## 3. Results

Excluded from the data analysis were four of the control group and one of the Oxytocin group, all for failure to attain comprehensive lab results (hemolytic sample, coagulated sample).

Demographic characteristics were comparable between the groups (Table 1).

In the Oxytocin group, the dose of administered Oxytocin ranged 2–16 mIU/min, (mean 9.3 mIU/min). Duration of treatment was between 1–11 hours (mean 3.7 hours). The sole indication for treatment was augmentation of labor.

**Figure 1:**
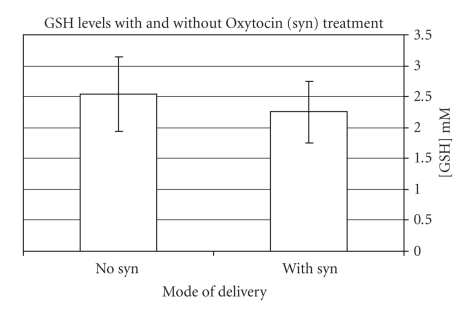
GSH level by Oxytocin treatment.

Cord blood GSH was significantly lower in the Oxytocin group (2.0 ± 0.55 mM versus 2.3 ± 0.55 mM, *P* = .01) (Figure 1).

In a multivariate analysis, adjusted for numbers of deliveries, fetal gender and fetal weight, Oxytocin remained the main predictor for GSH (Beta = (−1.859), *P* = .066).

Uptake of phenol red was similar between the groups (0.08 versus 0.076, *P* = .11). No differences were found between the groups in levels of MDA in plasma (0.9 ± 0.03 *μ*M versus 0.97 ± 0.05 *μ*M, *P* = .1) or MDA levels in RBC (2.05 ± 0.16 *μ*M versus 1.93 ± 0.10 *μ*M, *P* = .23).

Cord blood gas characteristics are summarized in Table 2.

Comparing the groups, significant differences were noted regarding length of delivery. First stage was significantly longer among the Oxytocin group (361 ± 267 minutes. versus 172 ± 130 minutes. *P* < .001) as was second stage of labor (25 ± 28 minutes. versus 13 ± 12 minutes. *P* = .01). Epidural anesthesia was more prevalent in the Oxytocin group (15% versus 4%, *P* = .05). No difference was noted in meconium stained amniotic fluid (15% versus 12%, *P* = .61) between the groups.

Comparison of GSH levels among first deliveries between the groups demonstrated a lower level of GSH following Oxytocin treatment (2.18 ± 0.40 mM versus 2.79 ± 0.17 mM, *P* = .002). The length of second stage of labor was similar in first deliveries in both groups (41 ± 38 minutes. versus 44 ± 25 minutes, *P* = .85).

As many as 63% of women from Oxytocin group were treated by iron supplements during pregnancy, while 72% received iron supplementation in the control group, *P* = .35. No correlation was found between iron treatment and GSH levels (correlation significance 0.73).

None received vitamin formulas during the last week before delivery. Two women from the Oxytocin group smoked during the pregnancy while none smoked in the control group, *P* = .1. The participants were questioned regarding disease prevalence. Gestational diabetes treated by diet alone was noted in two women from the Oxytocin group and in none from the control group, *P* = .1. No hypertensive disorders were detected. None had G6PD deficiency.

Obstetric history was examined for detection of high-risk pregnancies. No significant differences were noted between the Oxytocin and the control group regarding previous preterm labor (0.07 ± 0.2 versus 0.1 ± 0.5, *P* = .47), previous early abortion (0.2 ± 0.5 versus 0.3 ± 0.7, *P* = .6), or previous cesarean section (0 ± 0 versus 0.07 ± 0.2, *P* = .07). No difference was found regarding prenatal care between the groups, the Oxytocin group 2.7 ± 0.5 (in a scale of 1–3, 1 = lack of care, 3 = complete prenatal care) and 2.5 ± 0.6 for the control group, *P* = .2.

## 4. Discussion

Oxytocin augmentation of labor is an acceptably safe modality of treatment. Lower Glutathione levels compared to normal vaginal delivery indicate oxidative stress, yet no fetal red blood cell damage is instituted. The RBC antioxidant systems suffice to prevent cell damage of lipid peroxidation or increased permeability of the RBC membrane. Higher levels of GSH that are noted in the newborn according to previous publications [11] may contribute to an enhanced defensive mechanism against oxidative stress in the neonate [12]. pH levels were similar between the groups, in accordance with previous publications [8].

Oxytocin dose tapering is determined by frequency of contractions and labor progression, thus, the same drug dose might induce a different frequency of contractions in different patients as well as dissimilar rates of oxidative stress. Consistent with the local protocol of treatment by Oxytocin, frequency of contractions was maintained at less than five contractions in ten minutes. Dose of oxytocin was adjusted as needed to attain this goal. Quantification of Oxytocin treatment for the purpose of precise comparison between the groups was of concern due to the short half life of this hormone and the debate whether the total dose administered during labor or the last dosage of treatment is the most accurate indicator of the Oxytocin impact. Provided that a drug with short term effect administered hours prior to a measured oxidative reaction which is quick and changing in nature has questionable effect upon the outcome, the last dose administered was chosen as the index of treatment.

Direct measurements of Oxytocin in maternal blood might have improved the precision of our results though others have failed to demonstrate a correlation between measured blood Oxytocin concentration and fetal pH [8, 13, 14].

In the Oxytocin group, primiparity was more prevalent. Length of second stage of labor among primiparas was not significantly different between the groups (41 ± 38 minutes. versus 44 ± 25 minutes, *P* = .85). These parameters support the observation that GSH was lower in the study group due to Oxytocin treatment and not due to longer first deliveries. There is some evidence that the detected impact of Oxytocin upon GSH levels might become substantial in compromised fetuses [15, 16].

The correlation between Oxytocin level and lower GSH is not supported by lower GSH levels found during elective cesarean deliveries compared to vaginal deliveries [17]; this inconsistency may evolve from higher oxidative stress caused by the operative delivery and anesthesia process and not attributed to the labor-induced Oxytocin level. A study comparing fetal oxidative stress following elective and emergent cesarean sections demonstrated higher oxidative stress in emergent cesarean deliveries (increased MDA levels); the author implied in this case that the mode of delivery was not the main attributor to oxidative stress but rather previous fetal condition [18]. This remains to be further explored.

In conclusion, Oxytocin treatment for augmentation of low-risk vaginal deliveries contributes to oxidative stress; however, no fetal cell damage is induced. Higher-power studies are required to further establish these findings.

## Figures and Tables

**Table 1:** Demographic characteristics.

	With Oxytocin (mean ± Standard deviation) *n* = 40	Without Oxytocin (mean ± Standard deviation) *n* = 53	*P*
Maternal age (years)	26.9 ± 5.6	28.5 ± 5.7	*P* = .18
Pregnancy order	2.9 ± 2	4.1 ± 2	*P* = .01
Delivery number	1.6 ± 2	2.6 ± 2	*P* = .03
Gestational age (weeks)	39.0 ± 1.4	39.5 ± 1.3	*P* = .09
Birth weight (g)	3207 ± 350	3308 ± 405	*P* = .202

**Table 2:** Cord blood characteristics.

	With Oxytocin (mean ± Standard deviation) *n* = 53	Without Oxytocin (mean ± Standard deviation) *n* = 40	*P*
pH	7.31 ± 0.06	7.33 ± 0.06	*P* = .15
pO_2_ mMHg	36 ± 17	35 ± 21	*P* = .83
pCO_2_ mMHg	40 ± 10	40 ± 9	*P* = .94
Base excess	(−5.2) ± 2.2	(−4.6) ± 2.1	*P* = .16

## References

[1] Yaacobi N, Ohel G, Hochman A (1999). Reactive oxygen species in the process of labor. *Archives of Gynecology and Obstetrics*.

[2] Fogel I, Pinchuk I, Kupferminc MJ, Lichtenberg D, Fainaru O (2005). Oxidative stress in the fetal circulation does not depend on mode of delivery. *American Journal of Obstetrics and Gynecology*.

[3] MacLennan A (2000). A template for defining a causal relationship between acute intrapartum events and cerebral palsy: international consensus statement. International Cerebral Palsy Task Force. *Australian and New Zealand Journal of Obstetrics and Gynaecology*.

[4] Yoon BH, Kim SW (1994). The effect of labor on the normal values of umbilical blood acid-base status. *Acta Obstetricia et Gynecologica Scandinavica*.

[5] Jones DP, Coates RJ, Flagg EW (1992). Glutathione in foods listed in the National Cancer Institute's Health Habits and History Food Frequency Questionnaire. *Nutrition and Cancer*.

[6] Sawas AH, Pentyala SN (2004). Evaluation of lipid peroxidation in red blood cells by monitoring the uptake of sucrose and phenol red. *Journal of Applied Toxicology*.

[7] Smith JG, Merrill DC (2006). Oxytocin for induction of labor. *Clinical Obstetrics and Gynecology*.

[8] Thorp JA, Boylan PC, Parisi VM, Heslin EP (1988). Effects of high-dose oxytocin augmentation on umbilical cord blood gas values in primigravid women. *American Journal of Obstetrics and Gynecology*.

[9] Cahill DJ, Boylan PC, O'Herlihy C (1992). Does oxytocin augmentation increase perinatal risk in primigravid labor?. *American Journal of Obstetrics and Gynecology*.

[10] Beutler E, Duron O, Kelly BM (1963). Improved method for the determination of blood glutathione. *The Journal of Laboratory and Clinical Medicine*.

[11] Buhimschi IA, Buhimschi CS, Pupkin M, Weiner CP (2003). Beneficial impact of term labor: nonenzymatic antioxidant reserve in the human fetus. *American Journal of Obstetrics and Gynecology*.

[12] Silberstein T, Mankuta D, Shames AI (2008). Neonatal blood is more resistant to oxidative stress induced by stable nitroxide radicals than adult blood. *Archives of Gynecology and Obstetrics*.

[13] Xenakis EM-J, Langer O, Piper JM, Conway D, Berkus MD (1995). Low-dose versus high-dose oxytocin augmentation of labor—a randomized trial. *American Journal of Obstetrics and Gynecology*.

[14] Merrill DC, Zlatnik FJ (1999). Randomized, double-masked comparison of oxytocin dosage in induction and augmentation of labor. *Obstetrics and Gynecology*.

[15] Robles R, Palomino N, Robles A (2001). Oxidative stress in the neonate. *Early Human Development*.

[16] Hracsko Z, Orvos H, Novak Z, Pal A, Varga IS (2008). Evaluation of oxidative stress markers in neonates with intra-uterine growth retardation. *Redox Report*.

[17] Paamoni-Keren O, Silberstein T, Burg A, Raz I, Mazor M, Saphier O (2007). Oxidative stress as determined by glutathione (GSH) concentrations in venous cord blood in elective cesarean delivery versus uncomplicated vaginal delivery. *Archives of Gynecology and Obstetrics*.

[18] Lurie S, Matas Z, Boaz M, Fux A, Golan A, Sadan O (2007). Different degrees of fetal oxidative stress in elective and emergent cesarean section. *Neonatology*.

